# Auditory Brainstem Response Wave I Amplitude Has Limited Clinical Utility in Diagnosing Tinnitus in Humans

**DOI:** 10.3390/brainsci12020142

**Published:** 2022-01-21

**Authors:** Katie Turner, Omid Moshtaghi, Neil Saez, Matthew Richardson, Hamid Djalilian, Fan-Gang Zeng, Harrison Lin

**Affiliations:** Department of Otolaryngology, Head & Neck Surgery, University of California Irvine, Irvine, CA 92697-5320, USA; katieturner01@gmail.com (K.T.); omoshtaghi@gmail.com (O.M.); nasaez11@gmail.com (N.S.); mlrichar@uci.edu (M.R.); hdjalili@uci.edu (H.D.); Harrison.lin@uci.edu (H.L.)

**Keywords:** tinnitus, auditory brainstem response, biomarker, human

## Abstract

Animal studies have discovered that noise, even at levels that produce no permanent threshold shift, may cause cochlear damage and selective nerve degeneration. A hallmark of such damage, or synaptopathy, is recovered threshold but reduced suprathreshold amplitude for the auditory brainstem response (ABR) wave I. The objective of the present study is to evaluate whether the ABR wave I amplitude or slope can be used to diagnose tinnitus in humans. A total of 43 human subjects, consisting of 21 with tinnitus and 22 without tinnitus, participated in the study. The subjects were on average 44 ± 24 (standard deviation) years old and 16 were female; a subgroup of 19 were young adults with normal audiograms from 125 to 8000 Hz. The ABR was measured using ear canal recording tiptrodes for clicks, 1000, 4000 and 8000 Hz tone bursts at 30, 50, and 70 dB nHL. Compared with control subjects, tinnitus subjects did not show reduced ABR wave I amplitude or slope in either the entire group of 21 tinnitus subjects or a subset of tinnitus subjects with normal audiograms. Despite the small sample size and diverse tinnitus population, the present result suggests that low signal-to-noise ratios in non-invasive measurement of the ABR limit its clinical utility in diagnosing tinnitus in humans.

## 1. Introduction

Normal auditory brainstem responses (ABRs) consist of five far-field potential peaks arising in the auditory periphery during the first 10–15 ms after a transient stimulus. The first peak, known as wave I, reflects the synchronized output arising in the auditory nerve. The ABR has been widely used in the clinic for a range of purposes, from universal screening of newborn hearing to differential diagnosis of auditory neuropathy. Recently, the ABR has been shown to identify cochlear synaptopathy, a newly discovered hearing disorder resulting from even moderate noise exposure [1,2]. Cochlear synaptopathy does not refer to outer hair cell damage, but rather to swollen synapses between the inner hair cells and auditory nerve fibers; this produces secondary nerve injury predominantly in the low-spontaneous-rate, high-threshold nerve fibers [3]. Because the nerve fibers responding to higher stimulus levels are disproportionately affected, animals with cochlear synaptopathy have normal wave I amplitude at or near thresholds but reduced wave I amplitude at higher stimulus levels [1,4,5,6]. As a result of this differential effect, the corresponding slope of the wave I growth function is also reduced.

Since cochlear synaptopathy has been suggested as a mechanism underlying tinnitus [7,8], the ABR may serve as an objective biomarker for tinnitus in humans. Indeed, two studies found significantly reduced wave I amplitude in human tinnitus sufferers [9,10]. This positive finding of reduced wave I amplitude is theoretically important because it establishes a link between cochlear neuropathy, which has been demonstrated in animals only, and tinnitus, which can be only subjectively reported by humans. However, both studies adopted stringent inclusion criteria, limited to relatively young (mean age 33–43 years) tinnitus sufferers with normal audiograms (≤20 dB HL from 125 Hz to 8 kHz) [9,10]. Because only 15% of tinnitus sufferers have normal thresholds [11,12] and the prevalence of tinnitus increases with age to 70 years [13,14], these stringent criteria would exclude up to 90% of the general tinnitus population. This context raises questions over whether the ABR wave I amplitude is sufficiently sensitive or specific to differentiate between the majority of human listeners with and without tinnitus.

The present study measured the ABR in a diverse group of tinnitus subjects and also in a control group of non-tinnitus subjects that was matched to the tinnitus group in age and hearing loss. The hypothesis was that if cochlear synaptopathy played a major role in humans as conjectured by animal studies, then the ABR wave I amplitude or slope should be reduced in the tinnitus subjects.

## 2. Materials and Methods

### 2.1. Subjects

A total of 43 human subjects participated in the present study. The control group consisted of 22 non-tinnitus subjects with a mean age of 44 ± 24 (standard deviation (SD)) years, including 8 females and 14 males, and a subgroup of 11 young adults with normal hearing (thresholds ≤ 20 dB HL from 125 Hz to 8 kHz). The experimental group consisted of 21 tinnitus sufferers with a mean age of 43 (±16 SD) years, including 8 females and 13 males, and a subgroup of 8 young adults with normal hearing. Extended audiometric test revealed moderate hearing loss at 12 kHz but no significant difference between the control and tinnitus groups (39 ± 31 vs. 48 ± 28 dB HL, *p* = 0.37). The 21 tinnitus subjects had an average tinnitus severity index score of 35 (SD = 19) [15] and a hyperacusis index of 34 (SD = 20) [16], with both indices being normalized to a 0–100 range. The suspected tinnitus etiology was noise (*n* = 13), trauma (*n* = 1), sudden hearing loss (*n* = 1), ear infection (*n* = 1), or unknown (*n* = 5). None of the tinnitus subjects reported Meniere’s disease, otosclerosis, auditory neuropathy, or acoustic neuroma that might affect the ABR morphology.

### 2.2. Auditory Brainstem Responses

**Figure 1:**
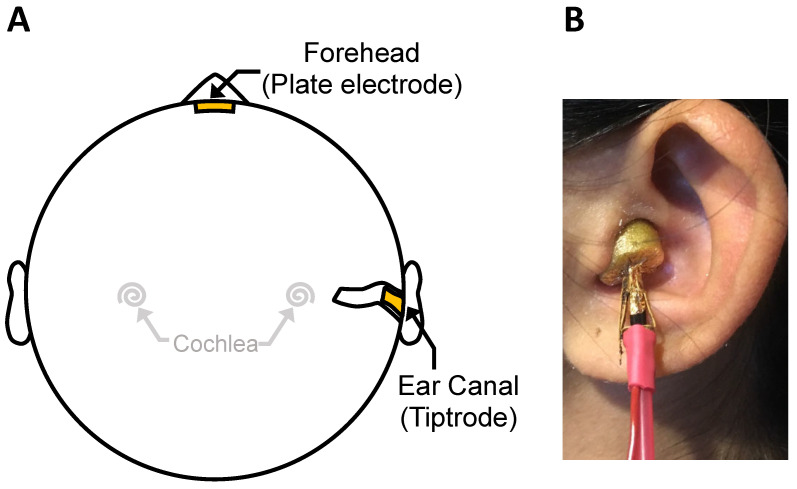
Experimental setup: (**A**) Electrode montage between a tiptrode inserted in the ear canal and a Jelly Tab plate electrode on the forehead. (**B**) Picture showing the gold-plated tiptrode and the sound delivery black tube.

The ABR was measured using an ear canal electrode for clicks and three tone bursts of 1000, 4000, and 8000 Hz, presented at three levels of 30, 50, and 70 dB nHL. The ear canal electrode was chosen for its sensitivity to the ABR wave I [17], whereas the additional three tone stimuli were used to limit the role of hearing loss. The subject was seated in a recliner and instructed to remain still and relax as much as possible in a double-walled, sound-attenuated booth. For subjects without tinnitus, the ear with better thresholds was tested. For subjects with unilateral tinnitus, the tinnitus ear was tested. For subjects with bilateral tinnitus, the ear with more severe tinnitus was tested (unless hearing loss or hyperacusis in that ear prevented effective testing, in which case the ear with less severe tinnitus was tested). The ABR was measured using the Bio-logic Auditory Evoked Potentials (AEP) system (version 6.2.1.1(d), Natus Medical Inc., Schaumburg, IL, USA). An ear canal and forehead montage (Figure 1A) was used, with a tiptrode being inserted in the ear canal (Figure 1B) and a Natus Jelly Tab Sensor plate electrode being placed on the forehead [17,18,19]. The skin area was prepared with alcohol and conductive gel such that impedance was ≤5 kOhms. The gold-plated tiptrode was also designed for sound delivery with ER-3 earphones (Etymotic Research Inc., Elk Grove Village, IL, USA). The stimuli comprised 100 μs clicks and 3 tone bursts (1000 Hz, 4000 Hz, and 8000 Hz). Tone bursts had a rise and fall time of 0.5 ms (Blackman window) and a plateau of 4 ms. All stimuli were presented at a rate of 10.3/s, with alternating polarity, and at 30, 50, and 70 dB nHL. These dB nHL values corresponded to 55, 75, and 95 dB peak sound pressure level (SPL), respectively, for a continuous 1000 Hz tone calibrated by a sound level meter. For each stimulus, at each sound level, 8000 repetitions were obtained to form an averaged ABR waveform. The recording epoch was 21.33 ms, beginning 5 ms prior to stimulus, with 512 sampling points recorded. Gain was set at 100,000 and the waveform was bandpass filtered between 3 and 5000 Hz. Any waveform with amplitude greater than 23.80 μV was rejected as artifact. The numbers of accepted and rejected waveforms were monitored during collection. In case of excessive artifact occurrences, which were usually caused by the subject’s movement, the subject was repositioned or given a break.

### 2.3. Data Analysis

The recorded ABR waveform was imported into Matlab (MathWorks, Natick, MA, USA) for additional off-line processing. Direct current drift was removed by subtracting a linearly fitted line based on individual epochs (detrend.m in Matlab). A waveform baseline was defined as the average of the 4 ms prior to stimulus presentation. The ABR wave I amplitude was defined as the difference between the wave I peak and following trough [10]. The wave I slope (μV/dB) was obtained by a linear fit of the wave I amplitude as a function of stimulus levels from 30 to 70 dB nHL.

Two-tailed, two-sample *t*-tests were used to compare the tinnitus and control ABR results. Both within-subjects and between-subjects analysis of variance (ANOVA) tests were performed to test for main stimulus and tinnitus effects and their interactions. The within-subjects factor was stimulus type, while the between-subjects factor was tinnitus. Multiple regression was used to further delineate relative contributions of various subject variables to the ABR wave I amplitude and slope in the tinnitus group.

## 3. Results

**Figure 2:**
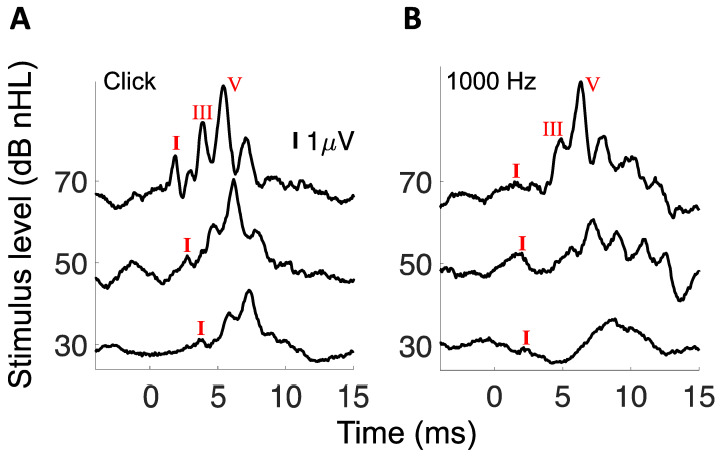
(**A**) Sample ABRs to clicks. (**B**) Sample ABRs to 1000 Hz tone bursts. Wave I was labeled for all waveforms while wave III and V are labeled for the 70 dB nHL stimulus only.

Figure 2 shows sample ABRs to clicks and the 1000 Hz tone burst.

Table 1 shows that in the present cohort of subjects, tinnitus subjects did not show reduced ABR wave I amplitude (columns under the left underlined, *p* = 0.178–0.863) or slope (columns under the right underlined, *p* = 0.182–0.797). The between-subjects ANOVA confirmed this insignificant effect of tinnitus on both the wave I amplitude (*F*(1,36) = 0.14, *p* = 0.72) and slope (*F*(1,38) = 0.55, *p* = 0.46). The within-subjects ANOVA showed a significant effect of stimuli on both the amplitude (*F*(1,36) = 51.30, *p* < 0.001) and slope (*F*(1,38) = 38.24, *p* < 0.001), with the click having the largest amplitude and slope values while the 1000 Hz tone burst having the smallest values. No significant tinnitus interaction was observed with either the amplitude (*F*(1,36) = 0.23, *p* = 0.64) or slope (*F*(1,38) = 0.04, *p* = 0.84), suggesting that the significant stimulus effect was primarily due to differences in stimulus properties and their evoked neural responses.

Additionally, multiple regression was performed using age, average threshold and tinnitus (no tinnitus = 0, tinnitus = 1) as independent variables to predict either the wave I amplitude or slope. For the 70 dB click, the age alone could account for 40.7% variance in the wave I amplitude (*F*(1,40) = 26.78, *p* < 0.001) or 26.3% variance in the slope (*F*(1,42) = 14.61, *p* < 0.001); no more significant amount of variance could be accounted for by adding either the average threshold or the tinnitus variable (*p* > 0.39). The reason for no additional contribution of the hearing threshold was that the threshold was highly correlated with the age (*r* = 0.83, *n* = 43, *p* < 0.001). On the contrary, tinnitus was not significantly correlated with either age (*r* = −0.04, *p* = 0.80) or the hearing threshold (*r* = 0.16, *p* = 0.31), suggesting that tinnitus itself did not make any difference in the wave I amplitude or slope. Finally, analysis was performed on a subset of 11 control and eight tinnitus young subjects who all had normal hearing. Even under this stringent condition, no significant difference was observed between the tinnitus and control groups for either the wave I amplitude or slope (*p* = 0.06–0.97, see Table 2).

## 4. Discussion

At first glance, the present negative result seemed to contradict the positive tinnitus effect observed by the two previous studies [9,10]. Schaette and McAlpine (2011) found smaller wave I amplitude in the tinnitus group than in the control group: 90 dB: 0.091 ± 0.009 vs. 0.12 ± 0.012 μV; 100 dB: 0.151 ± 0.015 vs. 0.203 ± 0.017 μV (*p* = 0.009, two-way ANOVA) [9]. They did not report any post-hoc test results. Assuming the value after “±” as the standard error and using the reported sample size of 18 for the tinnitus group and 15 for the control group, two-tailed, two-sample *t*-test without correction produced an insignificant tinnitus effect on the 90 dB condition (*t* = –2.02, *p* = 0.053) but a significant effect on the 100 dB condition (*t* = –2.29, *p* = 0.029). Gu et al. (2012) reported (from their Table 3) the following wave I amplitude values between their tinnitus and control groups: 70 dB nHL: 0.18 ± 0.02 (*n* = 25) vs. 0.21 ± 0.02 μV (*n* = 23); 80 dB nHL: 0.20 ± 0.03 (*n* = 11) vs. 0.33 ± 0.03 μV (*n* = 14) [10]. The same *t*-test produced the same result: insignificant effect on the 70 dB condition (*t* = –1.07, *p* = 0.29) but significant on the 80 dB condition (*t* = –3.05, *p* = 0.006).

Therefore, the present negative result at 70 dB nHL was consistent with the insignificant effect at 90 dB SPL in one study [9], and at the 70 dB nHL in the other [10]. Regardless of recording devices, measuring ABR at 80 dB nHL or higher levels is not always practical. Two of the 21 tinnitus subjects in the present study did not even participate in the 70 dB nHL condition because they could not tolerate its loudness due to self-reported hyperacusis. Gu et al. (2012) encountered the same problem as their number of tinnitus subjects decreased from 25 at 70 dB to 11 at 80 dB nHL [10]. Indeed, 40–50% tinnitus sufferers have co-morbid hyperacusis [20,21], preventing the measurement of ABR at high levels. Alternative methods must be employed to translate the ABR measure in animals into a tinnitus biomarker in humans.

One method is to increase the ABR signal-to-noise ratio in humans. ABRs obtained with invasive subdermal electrodes in animals have much higher signal-to-noise ratios than with non-invasive scalp electrodes in humans. For comparison, Kujawa and Liberman needed only 512 averages to obtain ~3 μV wave I amplitude for tone bursts presented at 90 dB SPL [1], whereas the present study used 8000 averages to only produce an order of magnitude smaller wave I amplitude (0.06–0.24 μV, see Table 1) for tone bursts and clicks presented at ~95 dB peak SPL. Although it is unlikely that invasive subdermal electrodes will be applied for this purpose in humans, electrodes resting on the tympanic membrane can increase wave I amplitude [22] and may be used to improve differentiation between tinnitus and non-tinnitus subjects.

Another method is to use different ABR measures. For example, wave V typically has higher amplitude than wave I, but wave V may have limited clinical utility since the wave V amplitude may decrease, increase, or remain unchanged in human tinnitus subjects [9,10,23]. Compared with ABR amplitude, ABR latency may be a better candidate biomarker for tinnitus. One reason latency could be useful is that the human cochlea and head are larger than commonly used laboratory animals such as mice, rats and cats, likely accentuating latency differences in humans. Moreover, latency is related to temporal processing impairment observed in human subjects with tinnitus [24,25]. The clinical utility of ABR latency as a biomarker for cochlear synaptopathy has recently been explored in humans [26].

The present study highlights major differences in methods between animal studies and their human translation into clinics. Animal studies can be conducted under the most ideal environment. For example, animal studies can accurately control not only subject variables such as age, sex, genetics, hearing, noise exposure, and even weight, but also stimulus variables such as movement (by using anesthesia) and signal-to-noise ratios (by using invasive electrode placement) [4]. In contrast, human studies cannot always control these variables and in fact have to take them into account in developing sensitive yet specific clinical diagnostic protocols. At present, tinnitus diagnosis relies on subjective reporting. Objective diagnosis of tinnitus is absent and especially challenging for tinnitus patients with comorbid hearing loss (~80% overlap) [11,12] or hyperacusis (~50% overlap) [20,21]. Tinnitus prevalence and severity may also interact with age, sex, noise exposure, and other lifestyle and environmental factors [27]. Although the present study is limited by the small sample size and the lack of control for subjects with hearing loss but no tinnitus, it shows the difficulty in translating an apparently significant ABR measure in animals into a clinically meaningful biomarker in human tinnitus sufferers.

## 5. Conclusions

To test the clinical utility of the ABR wave I amplitude or slope in diagnosing tinnitus, the present study systematically measured the auditory brainstem response (ABR) to clicks, 1000, 4000, and 8000 Hz tones at 30, 50, and 70 dB nHLs, in a diverse group of 43 human subjects with (*n* = 21), and without (*n* = 22), tinnitus. Tinnitus did not reduce the wave I amplitude or slope for any of the four stimuli, even when the potentially confounding hearing loss factor was removed. The present result suggests that the clinical utility of conventional ABR measurement is limited in detecting tinnitus in humans.

## Figures and Tables

**Table 1:** Auditory brainstem responses wave I amplitude and slope from 22 non-tinnitus control and 21 tinnitus subjects.

		Wave I Amplitude at 70 dB nHL (μV)			Wave I Amplitude Slope (μV/dB)		
Stimulus	Tinnitus	Mean	95% CI	*p* Value	Mean	95% CI	*p* Value
Click	No	0.2192	0.1483−0.2901	0.66	0.0040	0.0022−0.0058	0.49
	Yes	0.2397	0.1841−0.2953		0.0048	0.0035−0.0061	
8000 Hz Tone	No	0.1111	0.0728−0.1494	0.86	0.0014	0.0004−0.0024	0.80
	Yes	0.1069	0.0794−0.1344		0.0016	0.0010−0.0022	
4000 Hz Tone	No	0.1413	0.1031−0.1795	0.18	0.0026	0.0016−0.0036	0.18
	Yes	0.1830	0.1374−0.2286		0.0037	0.0025−0.0049	
1000 Hz Tone	No	0.0614	0.0475−0.0753	0.45	0.0007	0.0002−0.0012	0.25
	Yes	0.0693	0.0548−0.0838		0.0010	0.0006−0.0014	

Confidence Interval, CI.

**Table 2:** Auditory brainstem responses wave I amplitude and slope from 11 non-tinnitus control and 8 tinnitus subjects who were young and had normal hearing.

		Wave I Amplitude at 70 dB nHL (μV)			Wave I Amplitude Slope (μV/dB)		
Stimulus	Tinnitus	Mean	95% CI	*p* Value	Mean	95% CI	*p* Value
Click	No	0.3452	0.2496−0.4408	0.81	0.0063	0.0033−0.0093	0.73
	Yes	0.3293	0.2442−0.4144		0.0069	0.0050−0.0088	
8000 Hz Tone	No	0.1633	0.1106−0.2160	0.78	0.0022	0.0005−0.0039	0.97
	Yes	0.1540	0.1205−0.1875		0.0023	0.0015−0.0031	
4000 Hz Tone	No	0.2114	0.1665−0.2563	0.07	0.0041	0.0028−0.0054	0.06
	Yes	0.2668	0.2090−0.3246		0.0061	0.0049−0.0073	
1000 Hz Tone	No	0.0776	0.0619−0.0933	0.06	0.0090	0.0067−0.0113	0.49
	Yes	0.0990	0.0855−0.1125		0.0100	0.0086−0.0114	

Confidence Interval, CI.

## Data Availability

The data presented in this study are available on request from the corresponding authors.
